# Cancer chemoresistance and BAK

**DOI:** 10.18632/oncoscience.276

**Published:** 2015-12-08

**Authors:** Joanna L. Fox

**Affiliations:** MRC Toxicology Unit, Hodgkin Building, Leicester, LE1 9HN, UK

**Keywords:** Apoptosis, BAK, chemotherapeutic resistance

Blocking or evading apoptosis is one of the hallmarks of cancer [1]. Recent advances in genomic sequencing have identified many mutations in cancer cells, prompting an increased effort to identify driver mutations in critical pathways that impact upon cell survival. Interestingly though, many cancers have intact apoptotic machinery where mutation rates of key regulators is very low - the BCL-2 family of proteins that regulate mitochondrial apoptosis being an important example. This highlights a key observation that cancer cells can prevent the initiation of apoptosis by mechanisms other than genomic mutation in key regulatory proteins, such as modifying epigenetic signatures, or by post-translational modifications - exemplified by a novel mechanism that we have discovered.

Chemotherapy remains the mainstay of treatment for most cancers. Paradoxically, the intrinsic or acquired resistance often displayed by cancer cells of different origin to chemotherapeutic agents that damage cells in disparate ways, coupled with drug-related toxicities, reduce drug efficacy and pose major barriers to effective treatment [2]. Treatment with cytotoxic agents triggers activation of the BCL-2 family of proteins, key regulators of cell death. This process depends on the activation of BCL-2 effector proteins, BAK and BAX, that exert their effects at a nodal commitment point in the apoptotic cascade by integrating apoptotic versus survival signals to determine cell fate [3]. Once an apoptotic ‘threshold’ has been overcome and sufficient BAK/BAX molecules have been activated, a cell becomes committed to die. Every cell has an apoptotic threshold, which is widely referred to as how ‘primed’ a cell is to die. Extensive studies of various types of cancer has revealed that the apoptotic threshold, although dependent on many factors such as the level of expression of anti-apoptotic proteins [4], is raised in cancer.

Our recent study published in Cancer Research [5] detailed a novel mechanism which contributes to determining how ‘primed’ a cancer cell is to die. The BCL-2 effector protein BAK is constitutively present in the outer mitochondrial membrane, and therefore requires tight regulation to ensure that aberrant apoptosis does not occur. Two key dephosphorylation events regulate the ability of BAK to undergo activation in response to death signals. The initial BAK dephosphorylation at Y108 by PTPN5 [6] is the key switch that enables BAK activation to proceed, and this is followed by dephosphorylation of S116 by PP2A [7]. Once these two phosphorylation marks have been removed then BAK activation can proceed as described in detail elsewhere [8].

**Figure 1:**
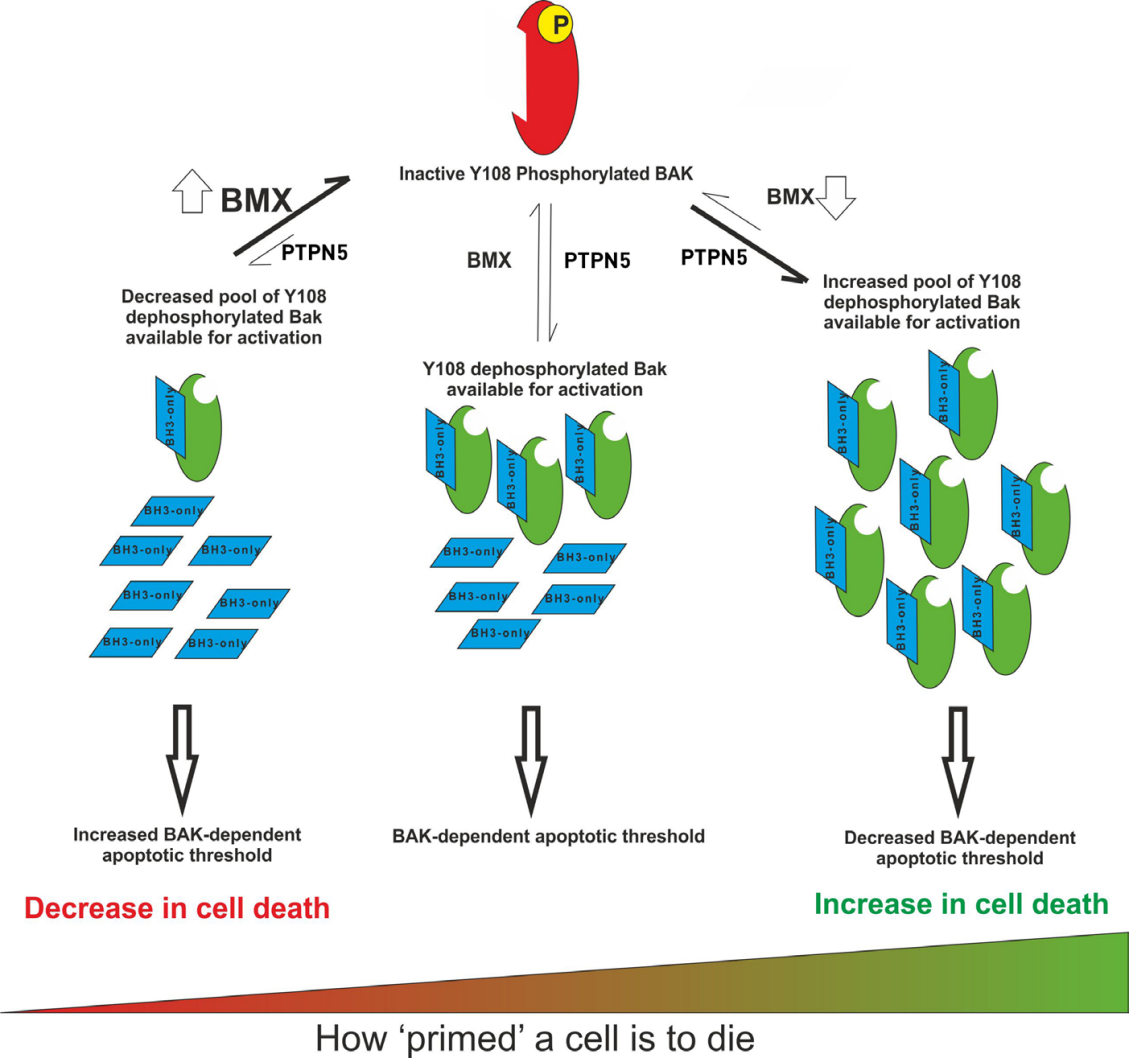
How BAK dephosphorylation sets the apoptotic threshold of the cell The size of the pool of Y108-dephosphorylated BAK contributes to determining a given cells sensitivity to undergo apoptosis, therefore setting the apoptotic threshold within that cell. Modulating BMX activity either suppresses or potentiates BAK activity that directly controls the levels of cell death.

We now show that the tyrosine kinase BMX, acting in concert with PTPN21, phosphorylates BAK on Y108 to suppress BAK activation to favor survival of cells exposed to cytotoxic agents (5; Figure 1). Importantly, we showed that when BMX activity was suppressed, BAK activity was potentiated and cancer cells became hyper-sensitive to a given dose of drug. Conversely, BMX over-expression maintained BAK in an inactive state and increased drug resistance. Up-regulation of BMX expression was found in many cancer types including prostate, breast and colon cancers. In cell lines, we find that BMX was over-expressed in triple-negative breast cancers, for which the only current treatment remains cytotoxic chemotherapy.

With increasing interest in targeting apoptotic proteins to re-activate apoptosis in cells with either intrinsic or acquired resistance to existing chemotherapeutic agents, targeting BMX represents a novel approach to directly modulate the level of apoptotic priming in the cell. The ability to make a cell more ‘primed to die’ has enormous clinical relevance, not only to increase the efficacy of current and emerging treatments, but also potentially to decrease side effects by lowering the dose of cytotoxic drugs patients require. Furthermore, BMX may also be an important biomarker for survival dependency in specific cancer types.
